# Dyeing of soybean protein/flax blended yarns with reactive dyes and subsequent dye-fixation

**DOI:** 10.1038/s41598-022-05581-5

**Published:** 2022-01-27

**Authors:** Jie Liu, Wenqi Jiang, Chun Lv

**Affiliations:** 1 College of Light-Industry and Textile, Qiqihar University, Qiqihar, 161006 China; 2 College of Architecture and Civil Engineering, Qiqihar University, Qiqihar, 161006 China

**Keywords:** Polymer chemistry, Supramolecular chemistry

## Abstract

The dyeing process of soybean protein/flax blended yarns with reactive dyes (containing monofunctional or bifunctional groups) and the method of improving the color fastness of dyed yarns treated with an ecofriendly formaldehyde-free fixing agent were studied. Influence factors such as sodium carbonate concentration, salt concentration, fixation time and temperature were analyzed, the optimum processes to soybean protein/flax blended yarns dyed with the two reactive dyes were determined: the soybean protein/flax blended yarns were dyed with Reactive Yellow K-R at a bath to material ratio of 20:1, dye concentration 2% owf., sodium chloride 40 g/L, sodium carbonate 10 g/L, fixed at 85 °C for 30 min; and dyed with Reactive Yellow B-4RFN at a bath to material ratio of 20:1, dye concentration 2% owf., sodium chloride 50 g/L, sodium carbonate 15 g/L, fixed at 70 °C for 50 min. The application processes of formaldehyde-free fixing agent DM-2158 were also determined. Performance test results indicated that both K-type and B-type reactive dyes had good colorfastness to washing and rubbing, and B-type reactive dyes showed a higher fixing effect than K-type reactive dyes. The application of a formaldehyde-free fixing agent to dyed products improved the colorfastness to washing and rubbing, especially for blended yarns dyed with K-type reactive dyes.

## Introduction

With the progress of society, consumers not only pursue beauty and practicality but also pay increasing attention to comfort and health. At present, the trend of the world textile industry is based on the ecological concept of developing new technology, new fibers and new products and paying attention to each production step. This is of great significance to the technological progress of the textile industry, the improvement of printing and dyeing product quality and environmental protection. In the past twenty years, great changes have taken place in the world textile industry. A number of nontraditional natural fibers, such as soybean protein fibers, and traditional natural fibers, such as flax fibers, are now attracting interest. Soybean protein fibers were mainly composited with other materials, for example, a highly heat-resistant and biodegradable textile size for textile waste repair has been made by modifying soybean protein with glycol^1^. Soybean protein crosslinked with cotton fabric (SPCCF) prepared by the reaction of carboxylic cotton fabric and soybean protein has been treated for medical protective textiles immobilized with drugs^2^. Flax fibers can also be blended with other fibers or modified, such as using chitosan and zinc oxide to modify flax, and cellulose-based multifunctional flax fabric was obtained^3^. NFBF-PLA (Natural Fiber Braided Fabric-Polylactic Acid) is polylactic acid (PLA) composited with braided flax fiber to enhance the mechanical properties of the composite material^4^. Linen/artificial glass woven fabrics were treated with methylene diphenyl diisocyanate (MDI) mixed resin to develop green composites^5^. To take advantage of the good performances of the two fibers, soybean/flax blended yarns and fabrics were produced. Flax fibers are composed of cellulose, they are of high strength, moisture absorption, fast and antibacterial but have stiff handle and poor elasticity^6,7^. Soybean protein fibers are comprised of 23–55% proteins, the rest being polyvinyl alcohol, they are of good performances, such as soft, glossy, warm and so on. They are very suitable for the production of functional underwear and summer clothing. The blended products can not only compensate for the limitations of flax fiber but also overcome the limitations of soybean protein fiber, making themselves suitable to obtain high-quality products. They can be spun into high count yarns, knitted into single- and double-side rib jacquard knitting fabrics, and made into garments after processing. The woven fabrics can be mainly made into casual suits and bedding, which has broad development prospects^8–10^. The blended yarns developed by Jinya Group were more than 20 tex, which had not been mass-produced in China or reported in other countries. At present, the product color is single, and the dyeing problem of blended yarns has not been fundamentally solved. The molecular composition and structure of flax fibers and soybean protein fibers are different, so the adsorption performance to dyestuff is different, and the sensitivity to temperature and pH value is also different. To produce colorful blended yarns and fabrics, the dyeing process must be studied. A series of studies have reported the treatment of flax by plasma at low temperature, grafting modification, using supercritical CO_2_ technology, treating flax fiber with liquid ammonia, NMMO or degrading chitosan to improve dyeing rate and other aspects^11–13^. To improve the dyeing performance of flax fibers, auxiliaries such as non-silicone oxygen bleaching stabilizer and activators have been used for pretreatment^14–16^. Based on the characteristics of soybean protein fibers and flax fibers, two types of reactive dyes containing monofunctional and bifunctional groups were selected [Reactive Yellow K-R and Reactive Yellow B-4RFN]^17,18^. Although reactive dyes can bond to fibers, some of them still undergo hydrolysis. Therefore, color-fixing treatment is needed after dyeing, but the hydrophilicity of the dyed products will also decrease after traditional fixing treatment^19–21^. Traditional fixing agents have been limited because they contain formaldehyde, which is not conducive to environmental protection and human health, so the selection of appropriate eco-friendly formaldehyde-free color-fixing agents is very important^22,23^. There were some new formaldehydeless fixing agents, such as cationic color fixer WLSPR, formaldehydeless fixing agent 6050, active formaldehydeless color fixer FR-2, formaldehydeless hydrophilic color fixer DM-2521, DM-2518, formaldehydeless color fixer Z and so on^24–26^. Based on the characteristics of the blended yarns and the selected reactive dyes, the formaldehyde-free fixing agent DM-2518 was selected in this study.

The purpose of this study was to optimize the dyeing process of soybean protein/flax blended yarns with reactive yellow K-R and reactive yellow B-4RFN by analyzing and comparing the dyeing properties of the two different types of reactive dyes, and to further improve the color fastness of dyed yarns by fixing the dyed yarn with the ecofriendly formaldehyde-free color fixing agent DM-2518. At the same time, the dyed samples were analyzed by infrared spectroscopy.

## Materials and methods

### Materials

Soybean protein/flax blended yarns (70:30) used for reactive dyeing were kindly provided by Qiqihar Jinya Group (China).

### Chemicals

All chemicals used in this study were laboratory reagent grade. Reactive Yellow K-R and Reactive Yellow B-4RFN were purchased from Tianjin Tiancheng Chemical Co. Ltd. (China). Sodium hydroxide, sodium chloride, sodium carbonate, hydrogen peroxide (30% w/w), sodium silicate, and urea were purchased from Tianjin Kaitong Chemical Reagent Co. Ltd. (China). The formaldehyde-free fixing agent DM-2518 was kindly provided by Dymatic Chemicals, Inc. (China).

### Bleaching of yarns

The soybean protein/flax blended yarns were bleached in a 250-mL Erlenmeyer flask in a water bath and at 90 °C for 60 min with a bath to material ratio of 20:1. The samples of soybean protein/flax blended yarn (5 g) were immersed in a 100 ml bleaching bath that contained 25 g/L H_2_O_2_, 5 g/L Na_2_CO_3_, 5 g/L urea, and 3 g/L Na_2_SiO_3_. After bleaching, the yarn samples were removed from the bleaching bath and then rinsed in hot and cold water. Finally, the bleached yarns were dried to be ready for dyeing.

### Dyeing of yarns

The bleached yarns were dyed using two types of reactive dyes, reactive yellow K-R (with monofunctional groups) and reactive yellow B-4RFN (with bifunctional groups). Dyeing was carried out using different technique parameters at a bath to material ratio from 10:1 to 50:1 [10:1, 20:1, 30:1, 40:1, 50:1]. The dye concentration was 2% owf. The initial temperature of dyeing was 40 °C. Half the amount of sodium chloride (NaCl) was added after dyeing 5 min, the remaining salt was added in another 15 min. Fixing temperature maintained was from 60 to 90 °C [60, 70, 75, 80, 85, 90 °C], fixing time was adjusted to be from 20 to 60 min [20, 30, 40, 50, 60 min], alkali (Na_2_CO_3_) concentration was from 5 to 25 g/L [5, 10, 15, 20, 25 g/L], salt concentration was from 10 to 60 g/L [10, 20, 30, 40, 50, 60 g/L]. Alkali was added in batches. After dyeing, the yarns were soaped in 2 g/L soap powder and 2 g/L Na_2_CO_3_, and the liquor to material ratio was 30:1.

### Exhaustion and fixation

The absorbance (A) of dyeing and soaping solutions was tested using a UV–Vis spectrophotometer (Wuxi Keda Instrument Factory, 754PC, China). The exhaustion (E) and fixation (F) of reactive dyes on yarns were calculated using Eqs. (1) and (2), respectively.

1 $${\text{E}} = 100\% - {\text{B}}/({\text{A}} \times 2) \times 100\%$$

where A and B are the absorbance at λ_max_ of the standard dye in the dye bath and of the residual dye after dyeing, respectively.

2 $${\text{F}} = {\text{E}} - {\text{D}}/({\text{C}} \times 2) \times 100\%$$

where C and D are the absorbance at λ_max_ of the standard dye in the soap bath and of the residual dye after boiling, respectively.

### Colorfastness tests

The yarn colorfastness to washing was measured according to the Chinese standard method (GB/T 3921–2008) with a Washable Fastness Tester (Wenzhou Darong Textile Standard Instrument Factory, YGH-DM, China). Yarn colorfastness to rubbing (dry and wet) was measured according to the Chinese standard method (GB/T 3920-2008) with an Abrasion Fastness Tester (Shanghai Jinghua Technology Instrument Co., Ltd, V571R, China). The breaking strength of the blended yarns was measured according to the Chinese standard method (GB/T 3916-2008) with a single yarn strength tester (Laizhou Electronic Instrument Co., Ltd, YG061, China).

## Results and discussion

### Factors affecting the dyeing performance of soybean protein/flax blended yarns

#### Fixation temperature

**Figure 1:**
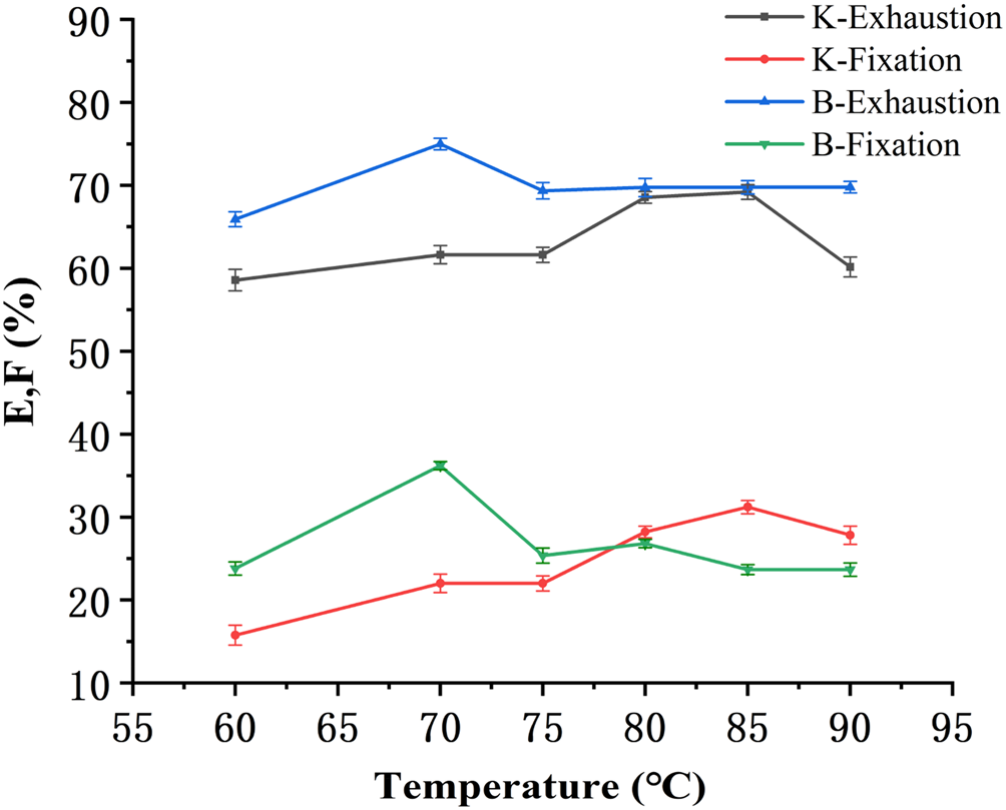
Relation between fixation temperature and E & F.

Fixation temperature has great influence on dyeing properties of the soybean protein/flax blended yarns. A higher temperature can swell the flax fibers and the soybean protein fibers, and promote adsorption, diffusion and penetration of the dyes. The bleached blended yarns were dyed at a bath to material ratio 20:1 with Reactive Yellow K-R (NaCl 40 g/L, Na_2_CO_3_ 10 g/L, fixed 30 min) and Reactive Yellow B-4RFN (NaCl 50 g/L, Na_2_CO_3_ 15 g/L, fixed 40 min). Exhaustion and fixation levels at different temperatures (60–90 °C) are shown in Fig. 1.

Figure 1 shows that Reactive Yellow B-4RFN achieved the maximum exhaustion and fixation levels at 70 °C, while Reactive Yellow K-R achieved it at 85 °C. At the beginning of fixation, with the increase of temperature, the extents of exhaustion and fixation of the soybean protein/flax blended yarns increased. But after reaching the maximum value, the exhaustion and fixation levels decreased gradually. The reason was that the soybean protein fibers in the blended yarns were not resistant to high temperatures, and under alkaline conditions, reactive dyes not only formed covalent bond with the fibers, but also occurred reverse hydrolysis when the temperature raised to a certain level. At the higher temperatures, the dyes bonded on the fibers hydrolyzed more quickly than their extent of fixation. So the fixation temperature should be at about 70 °C for Reactive Yellow B-4RFN and 85 °C for Reactive Yellow K-R.

#### Salt concentration

**Figure 2:**
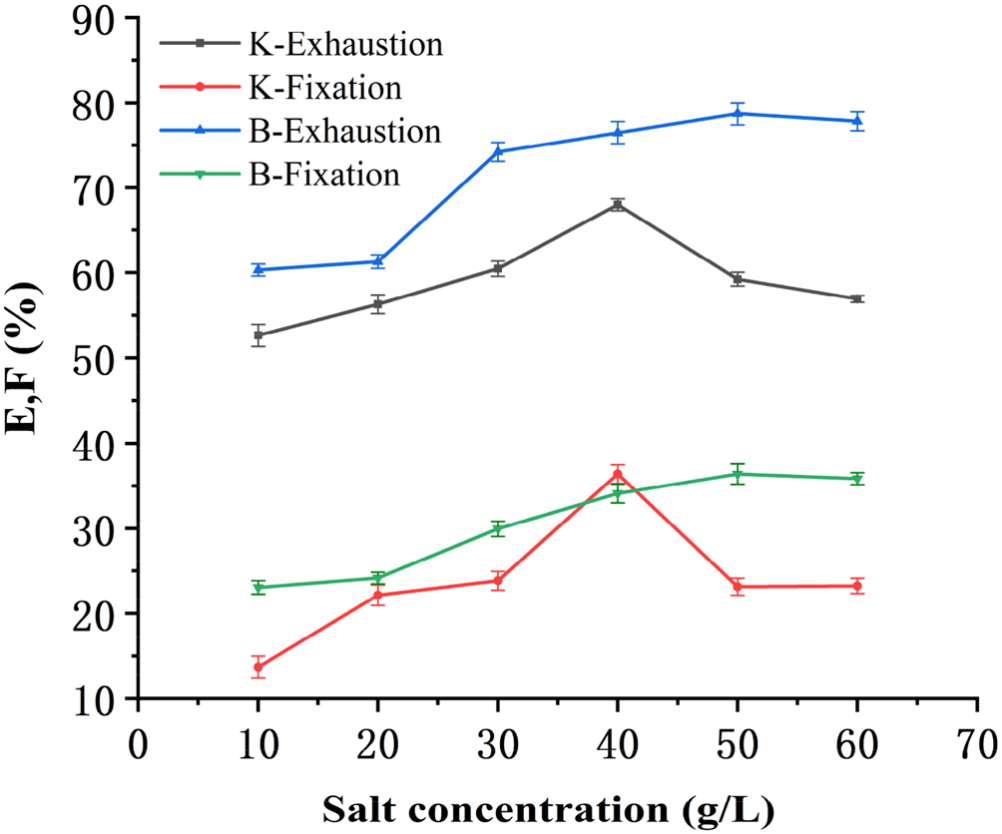
Relation between salt concentration and E & F.

In reactive dyeing, the function of neutral salt was to promote dyeing as an accelerant. Sodium chloride was the most common neutral salt, and the concentration of salt was related to the concentration of the dyes and liquor ratio and so on. The bleached blended yarns were dyed at a bath to material ratio 20:1 with Reactive Yellow K-R (Na_2_CO_3_ 10 g/L, fixed at 85 °C for 30 min) and Reactive Yellow B-4RFN (Na_2_CO_3_ 15 g/L, fixed at 75 °C for 40 min). Exhaustion and fixation levels at different salt concentrations (10–60 g/L) are shown in Fig. 2.

Figure 2 shows that for Reactive Yellow K-R, with the increase of the salt concentration, the exhaustion and fixation levels gradually increased, but after the amount of sodium chloride reached 40 g/L, the exhaustion and fixation levels decreased significantly. As for Reactive Yellow B-4RFN, the best salt concentration was 50 g/L sodium chloride. Meanwhile, at the maximum fixation level, the salt concentration for Reactive Yellow B-4RFN was higher than that of Reactive Yellow K-R, the reason was that bifunctional reactive dyes consumed more accelerating agent. However, an overdose of sodium chloride would cause uneven dyeing due to dye aggregation. So the sodium chloride concentration was about 40 g/L for Reactive Yellow K-R and 50 g/L for Reactive Yellow B-4RFN.

#### Alkali concentration

**Figure 3:**
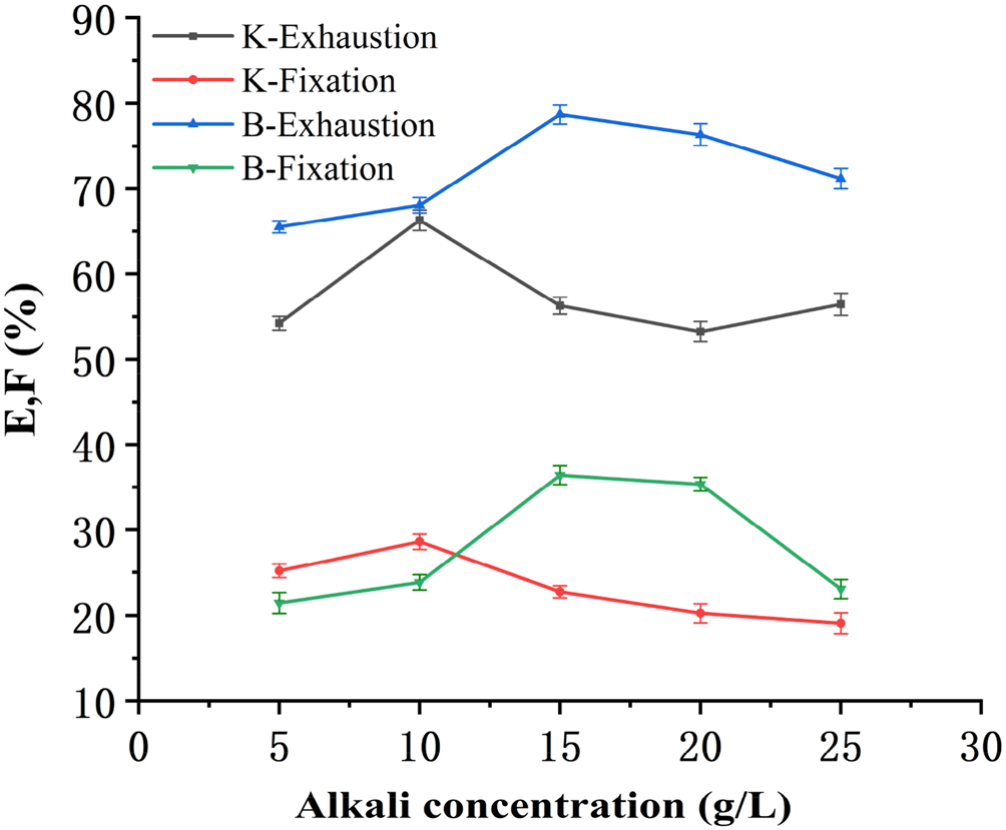
Relation between alkali concentration and E & F.

Under the alkaline condition of a certain pH value, the hydroxyl groups in the flax cellulose would form hydroxyl anions, and the amino groups in the soybean protein would form imino groups. The reactive dyes can react with the fibers and fix on the two fibers. The dyeing effect was related to the alkali concentrations and pH values of the dye bath. The strong alkali can lead to incomplete reaction with the fibre due to its hydrolysis, the bonding combination of the reactive dyes and fibers will not form firm, and to result resulting in a lighter color as well as uneven dyeing. Excessive alkali may cause the hydrolysis of the dyes, reduce the color yield, and decrease the fixation level. So sodium carbonate was selected in this study. The bleached blended yarns were dyed at a bath to material ratio 20:1 with Reactive Yellow K-R (NaCl 40 g/L, fixed at 85 °C for 30 min) and Reactive Yellow B-4RFN (NaCl 50 g/L, fixed at 75 °C for 40 min). Exhaustion and fixation levels at different alkali concentrations (5–25 g/L) are shown in Fig. 3.

Figure 3 shows that for Reactive Yellow K-R and Reactive Yellow B-4RFN, with the increase of the concentration of alkali, the exhaustion and fixation levels gradually increased, after reaching the maximum value, the exhaustion and fixation levels decreased when the concentration of alkali was further increased. The trend of the change in exhaustion and fixation levels was similar, but the optimal amount of alkali was different, sodium carbonate was required 10 g/L and 15 g/L for the two dyes, respectively. So the sodium carbonate concentration was 10 g/L for reactive yellow K-R and 15 g/L for reactive yellow B-4RFN.

The manner of adding alkali must be paid due attention. If the whole total quantity of sodium carbonate is added at one time, it will cause uneven dyeing, which implies that the flax fibers and the soybean protein/flax fibers would not achieve the same color. Pre-sharpen dyeing solved the uneven dyeing problem, in which case 1/3 quantity of sodium carbonate was added in the heating stage, and the rest 2/3 of sodium carbonate was added at the fixation temperature^27^. Reactive Yellow B-4RFN required 0.5–1 times more alkali than that required for Reactive Yellow K-R.

#### Fixation time

**Figure 4:**
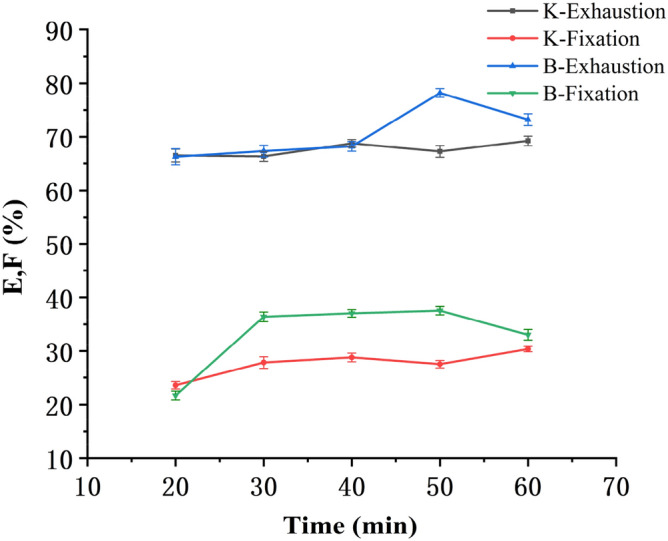
Relation between fixation time and E & F.

The bleached blended yarns were dyed at a bath to material ratio 20:1 with Reactive Yellow K-R (NaCl 40 g/L, Na_2_CO_3_ 10 g/L, fixed at 85 °C) and Reactive Yellow B-4RFN (NaCl 50 g/L, Na_2_CO_3_ 15 g/L, fixed at 75 °C). Exhaustion and fixation levels at different fixation times (10–60 min) are shown in Fig. 4.

As can be seen from Fig. 4, with the extension of the fixation time, both the exhaustion and fixation levels increased in different extents. It took 50 min for Reactive Yellow B-4RFN (30 min for Reactive Yellow K-R) to achieve the maximum exhaustion and fixation levels. During the dyeing, along with their reaction with the fibers, the hydrolysis reaction of the dyes also occurred, and produced the hydrolysed dyes with lower affinity to the fibers, which decreased their reactivity i.e. formation of covalent bonds between the dyes and fibers. So the fixation time was 50 min for Reactive Yellow B-4RFN and 30 min for Reactive Yellow K-R.

#### Bath to material ratio

**Figure 5:**
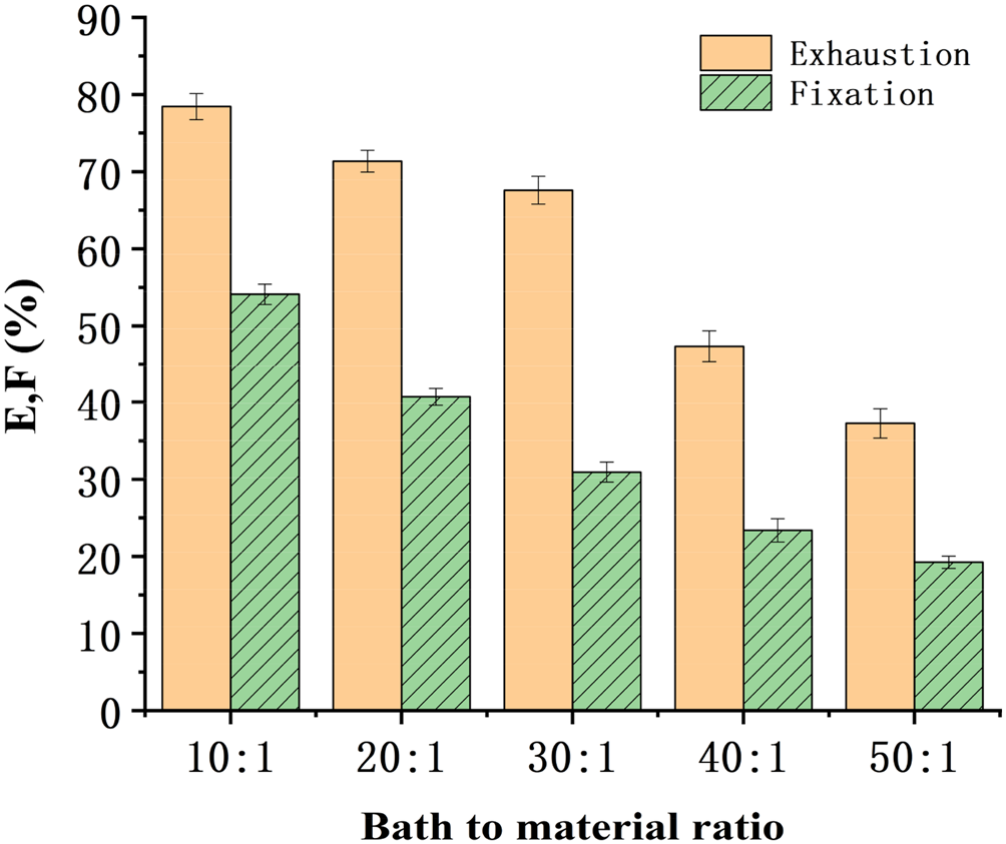
Relation between bath to material ratio and E & F (reactive yellow K-R).

**Figure 6:**
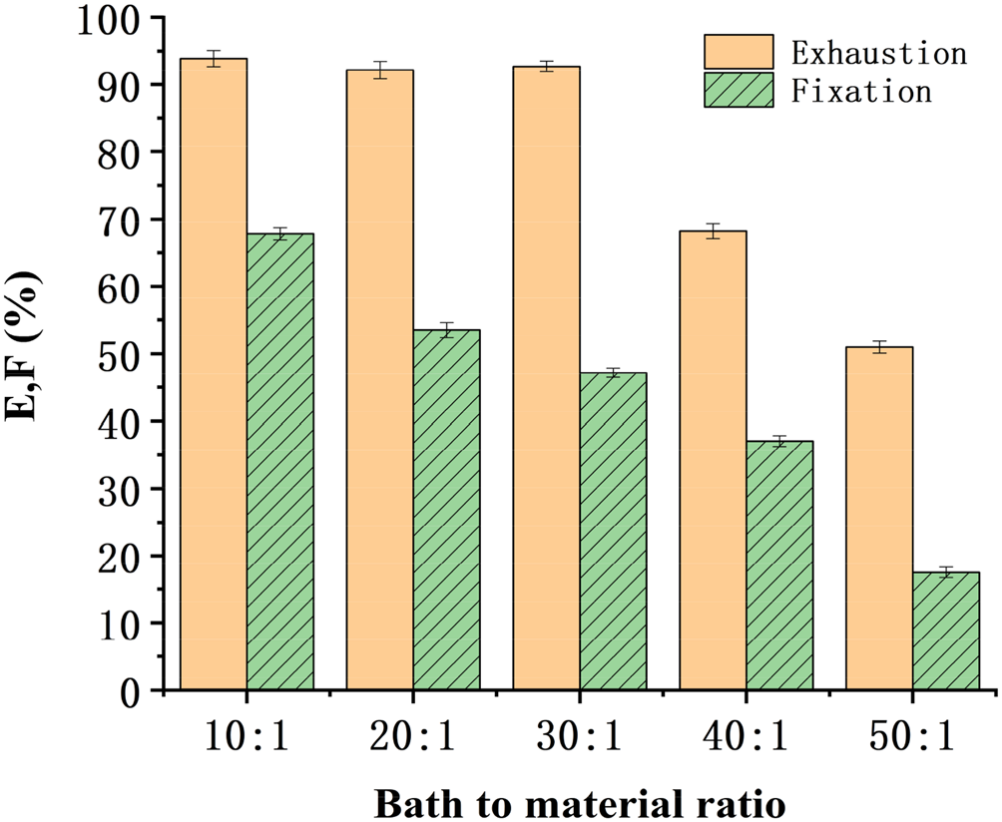
Relation between bath to material ratio and E & F (reactive yellow B-4RFN).

After changing the bath to material ratio, the bleached blended yarns were dyed with Reactive Yellow K-R (salt 40 g/L, alkali 10 g/L, fixed at 85 °C for 30 min) and Reactive Yellow B-4RFN (salt 50 g/L, alkali 15 g/L, fixed at 75 °C for 50 min). Exhaustion and fixation levels at different bath to material ratios are shown in Figs. 5 and 6.

Figures 5 and 6 show that the bath to material ratio significantly influenced the level of fixation of dyes, and the fixation level obviously increased with decreasing bath to material ratio. However, the bath to material ratio cannot be reduced indefinitely since a bath to material ratio lower than 10:1 in laboratory conditions significantly caused unevenness of dyeing, necessitating increased number of washing cycles. In the experiment, the dyed and soaped yarns under a bath to material ratio of 10:1 were washed with 30 ml water at least 5 times, and the pH value of the yarn surface was neutral. When the bath ratio was more than 30:1, the pH value of the yarn surface could reach neutral only after washing 2–3 times. However, a high bath to material ratio greater than 30:1 obviously decreased the fixation level and increased the consumption of other auxiliaries, which further increased the production cost. In factory production, the bath to material ratio could be reduced to 10:1 even lower according to the equipment conditions, but it must be pad or roll dyeing. However, due to the limitation of laboratory conditions, the bath to material ratio could be controlled to 20:1.

### Application of formaldehyde-free dye-fixing agent

To improve the color fastness of the dyed soybean protein/flax blended yarns, formaldehyde-free dye-fixing agent DM-2518 was selected to process the dyed yarns. DM-2518 is a water-soluble cationic polymer, the cations in its molecule can generate electrostatic bonding with the dye anion, the dyes with negative charge and the dye-fixing agents with positive charge can form insoluble color precipitation on fibers, so as to reduce the water solubility of the dye, and improve the wet treatment fastness.

### Factors affecting the colorfastness of soybean protein/flax blended yarns

#### Concentration of dye-fixing agent

The dyed soybean protein/flax blended yarns were treated with formaldehyde-free dye-fixing agent DM-2518, and the fastness to washing, rubbing and breaking strength of the dyed blended yarns were tested. The test results are shown in Table 1.

**Table 1:** Effect of dye-fixing agent concentration on colorfastness and breaking strength.

DM-2518 concentration (% owf.)	Fastness to washing (grade)		Fastness to dry rubbing (grade)		Fastness to wet rubbing (grade)		Breaking strength (cN)	
	K-R	B-4RFN	K-R	B-4RFN	K-R	B-4RFN	K-R	B-4RFN
1.0	3–4	3	4	3–4	3	3–4	321 ± 9	338 ± 9
1.5	4	3–4	4–5	4	3–4	4	321 ± 8	326 ± 8
2.0	4–5	4–5	5	4–5	4	4–5	319 ± 8	301 ± 7
2.5	4–5	4	5	4–5	4–5	4	307 ± 7	285 ± 6
3.0	3	3	4–5	4–5	4–5	4	289 ± 6	261 ± 5

Bath to material ratio 10:1, impregnating temperature 45 °C, time 20 min; curing 115 °C × 1 min.

It can be seen from Table 1 that for Reactive Yellow K-R, the optimum concentration of dye-fixing agent was 2.0% owf., which was the best of all colorfastness values and the optimum breaking strength retention. For Reactive Yellow B-4RFN, although the concentration of dye-fixing agent was 2.0% owf., it showed the best fastness to washing and rubbing, but the breaking strength of the blended yarns decreased greatly, therefore, the concentration of the dye-fixing agent DM-2518 was chosen as 1.5% owf..

#### Curing temperature

As the soybean protein fibers were not resistant to high temperature above 120 °C, the blended yarns were seriously damaged, especially for the soybean protein components in the blended yarns. Therefore, it was necessary to determine the appropriate curing temperature of the dye-fixing agent DM-2158 for the blended yarns. As shown in Table 2, cured at 115 °C, the color fastness to washing and to rubbing were better, the breaking strength of the blended yarn also decreased to a limited extent, hence the curing temperature was selected as 115 °C.

**Table 2:** Effect of curing temperature on colorfastness and breaking strength.

Curing temperature (°C)	Fastness to washing (grade)		Fastness to dry rubbing (grade)		Fastness to wet rubbing (grade)		Breaking strength (cN)	
	K-R	B-4RFN	K-R	B-4RFN	K-R	B-4RFN	K-R	B-4RFN
110	4	3–4	3–4	3–4	3	3	319 ± 7	324 ± 8
115	4–5	4–5	4	4	4	4	318 ± 7	319 ± 7
120	4	4–5	2–3	3	2	2	305 ± 5	273 ± 4

Bath to material ratio 10:1, impregnating 45 °C × 20 min; DM-2518 1.5% owf.; curing 1 min.

#### Curing time

The curing time was changed on the basis of the specific optimum curing temperature and the dye-fixing agent concentration. After the treatment, the performance indices of the blended yarns were tested. As shown in Table 3, when cured for 1.5 min for samples dyed with Reactive Yellow K-R and cured for 1 min for samples dyed with Reactive Yellow B-4RFN, the colorfastness to washing and rubbing was much better. Extending the curing time decreased the breaking strength of the yarns and the colorfastness. Therefore, the optimum curing times were 1.5 min and 1 min, respectively.

**Table 3:** Effect of curing time on colorfastness and breaking strength.

Curing time (min)	Fastness to washing (grade)		Fastness to dry rubbing (grade)		Fastness to wet rubbing (grade)		Breaking strength (cN)	
	K-R	B-4RFN	K-R	B-4RFN	K-R	B-4RFN	K-R	B-4RFN
1	4–5	4–5	4	4	3–4	4	317 ± 7	322 ± 7
1.5	5	4	4	3–4	4	3–4	316 ± 6	307 ± 5
2	3–4	3	3	3	3–4	3	302 ± 4	280 ± 4

Bath to material ratio 10:1, impregnating 45 °C × 20 min; curing 115 °C; DM-2518 1.5% owf.

### Dyeing enhancement

The soybean protein/flax blended yarns were dyed under the above mentioned optimized process conditions and the dye was applied 2, 4, and 6% owf., respectively. Then, the exhaustion and fixation levels were tested, the results are shown in Table 4.

**Table 4:** Effect of dye concentration on dyeing with the two reactive dyes.

Dye concentration (% owf.)	Reactive yellow K-R		Reactive yellow B-4RFN	
	E (%)	F (%)	E (%)	F (%)
2	82.85 ± 0.78	39.12 ± 0.53	86.64 ± 0.81	46.04 ± 0.63
4	74.49 ± 0.63	50.42 ± 0.62	78.21 ± 0.73	41.62 ± 0.54
6	65.27 ± 0.52	32.07 ± 0.54	57.03 ± 0.64	25.03 ± 0.47

**Figure 7:**
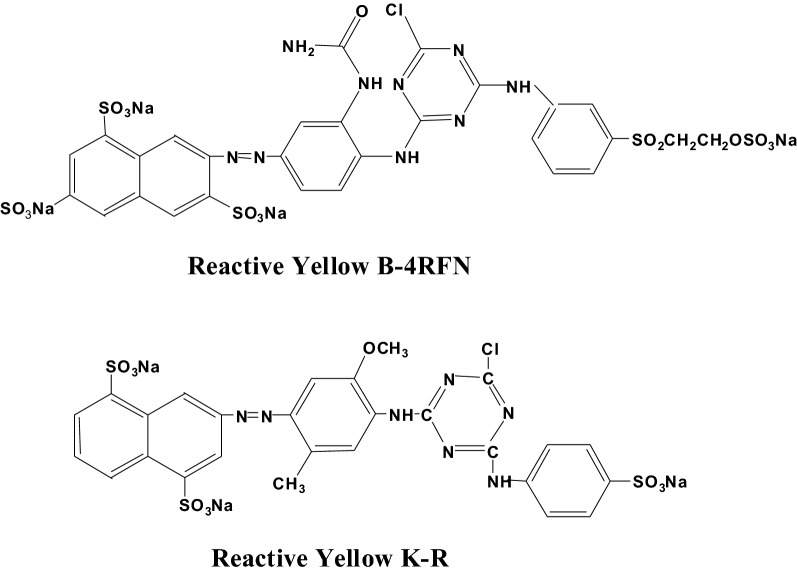
Chemical structure of the dyes.

Table 4 shows that for Reactive Yellow K-R, the dye increased from 2 to 4% owf., the fixation level increased from 39.12 to 50.42%, indicating that it had showed good enhancement in this range of dye concentration, and the dyeing depth could be improved by increasing the dye concentration. However, when the dye increased from 4 to 6% owf., the fixation level decreased from 52.42 to 32.07%, indicating that it was not suitable for deep shades, but more suitable for medium to light shades. K type reactive dye working solution is stable, in addition to react with fibers, but also combined with the fixing agent, so it showed better color fastness. For Reactive Yellow B-4RFN, the fixation level showed a downward trend as the dye concentration increased. The effect was related to the chemical constitution and structure of the dyes. Colour index numbers and chemical constitution and molecular weight: Reactive Yellow K-R (C.I. Reactive Yellow 3, C_27_H_19_ClN_7_Na_3_O_10_S_3_, molecular weight 802.090). Reactive Yellow B-4RFN (C.I. Reactive Yellow 145, C_28_H_20_ClN_9_Na_4_O_16_S_5_, molecular weight 1026.225). The chemical structure of these two dyes is shown in Fig. 7. Reactive Yellow B-4RFN is a dye with bifunctional groups, the ethylene sulfone group of B type reactive dye has a large space blocking effect on monochlorotriazine, so ethylene sulfone is the main reaction group when Reactive Yellow B-4RFN reacted with fiber macromolecules. However, vinyl sulfone sulfate must firstly generate vinyl sulfone group in alkaline condition, then combine with dye-fixing agent through double bond addition reaction, so its fixation effect was not very obvious. The dye that goes up adsorbed first prevents the dye that is adsorbed later from binding to with the fiber. So it was more suitable for dyeing light shades.

### Comparison of the dyeing performance

**Figure 8:**
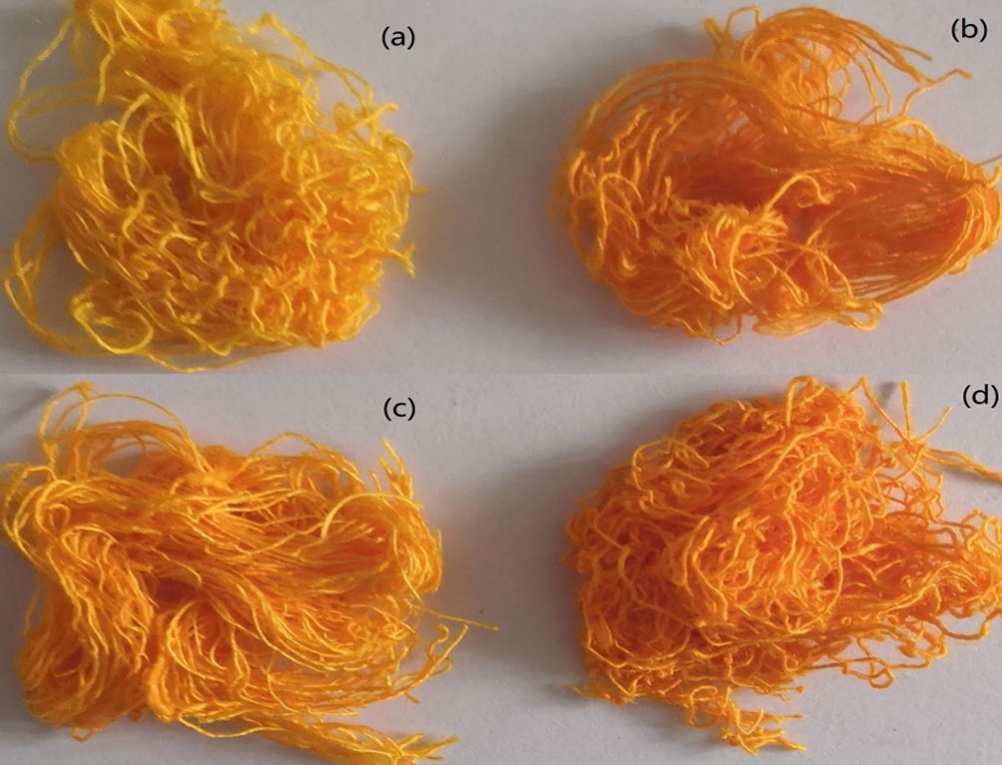
Photo of samples. (**a**) The blended yarns dyed with reactive yellow K-R; (**b**) the blended yarns dyed with reactive yellow B-4RFN; (**c**) the blended yarns dyed with reactive yellow K-R and fixed with DM-2158; (**d**) the blended yarns dyed with reactive yellow B-4RFN and fixed with DM-2158.

The soybean protein/flax blended yarns were dyed with Reactive Yellow K-R and Reactive Yellow B-4RFN under the optimum conditions, treated with formaldehyde-free color-fixing agent DM-2518, and then tested for colorfastness and breaking strength. A photo of the dyed (dyed and fixed) samples is shown in Fig. 8. The performance test results are shown in Table 5.

**Table 5:** Dyeing performance of dyed (dyed and fixed) samples.

Samples (dye 2% owf.)	E (%)	F (%)	Breaking strength (cN)	Fastness to washing (Grade)	Fastness to rubbing (grade)	
					Dry	Wet
Reactive yellow K-R (dyed)	82.85 ± 0.78	39.12 ± 0.48	326 ± 7	3	3	2–3
Reactive yellow B-4RFN (dyed)	84.64 ± 0.83	45.04 ± 0.52	342 ± 8	3–4	3–4	3
Reactive yellow K-R (dyed and fixed)	–	–	317 ± 6	5	4–5	4
Reactive yellow B-4RFN (dyed and fixed)	–	–	320 ± 6	4–5	4–5	4

Table 5 and Fig. 8 show that the exhaustion and final fixation levels of Reactive Yellow B-4RFN were significantly greater than those of Reactive Yellow K-R, as was the color fastness to washing and rubbing. This was mainly related to the fact that the bifunctional groups in Reactive Yellow B-4RFN can better form bonds with the fibers. It also indicated that both K-type and B-type reactive dyes had good colorfastness and levelness, especially for light to moderate shades, but B-type reactive dyes were better than K-type reactive dyes. The colorfastness of the dyed yarns can meet general requirements. Table 5 also shows that the color fastness to washing and rubbing improved obviously after treatment with the formaldehyde-free dye-fixing agent DM-2518, and the strength of the blended yarns was affected only slightly. This dye-fixing agent was especially suitable for K-type reactive dyes. The reason was that ethylsulfone sulfate in the molecule of B-type reactive dyes had a large spatial blocking stearic hindrance on monotriazine, and its reaction with fiber macromolecule was mainly through vinyl sulfone sulfate. The vinyl sulfone sulfate must form ethylsulfone group under alkaline condition through active double bond addition reaction to react with the fixing agent and hence its fixation effect was not obvious in B-Type reactive dye. K-type reactive dyes were stable, and in addition to reacting with the fiber when curing, they also combine with the fixing agent resulting in improved color fastness.

### Infrared spectra analysis

**Figure 9:**
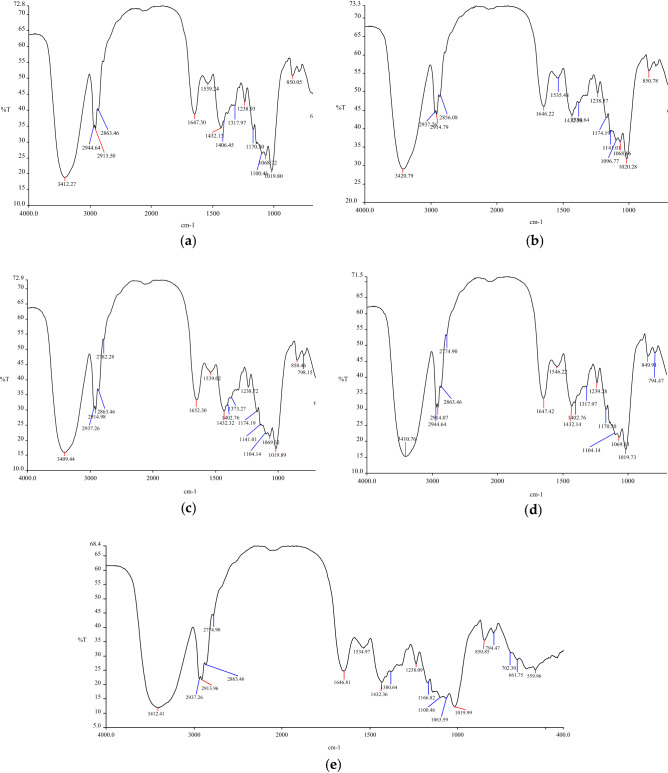
Infrared spectrum. (**a**) The undyed blended yarns; (**b**) the blended yarns dyed with reactive yellow K-R; (**c**) the blended yarns dyed with reactive yellow B-4RFN; (**d**) the blended yarns dyed with reactive yellow K-R and fixed with DM-2158; (**e**) the blended yarns dyed with reactive yellow B-4RFN and fixed with DM-2158.

To explore the combination of reactive dyes and blended fibers, the infrared spectra of soybean protein/flax blended yarn dyed with two reactive dyes were analysed and compared with the infrared spectra of the original yarn before dyeing. A Spectrum One Fourier Transform Infrared Spectrometer by PerkinElmer (USA) was used. The infrared spectrum of the samples is shown in Fig. 9.

In Fig. 9a, the peak observed at 3412.27 cm^−1^ was due to the –OH stretching vibration absorption peak and bending vibration absorption peak in the flax fiber. At 1019.8 cm^−1^ was due to the -NH_2_ vibration absorption peak in soybean protein fiber. In Fig. 9b, after dyeing with K-type reactive dye, the absorption peak at 3420.79 cm^−1^ was the covalent bending vibration absorption peak of cellulose -OH and homotriazine, and the absorption peak at 1020.28 cm^−1^ was the covalent bending vibration peak of amino groups in soybean protein and primary hydroxyl groups in PVA and homotriazine. In Fig. 9c, the two maximum absorption peaks were the cellulose hydroxyl group binding to the homotriazine and ethylene sulfone groups and the primary hydroxyl group in the amino PVA of soybean protein binding to the homotriazine and ethylene sulfone groups. After dyeing, the two absorption peaks of the product were subtracted from the two absorption peaks of the original yarn. The absorption peak of the product changed greatly after dyeing with K-type dye, while the absorption peak of the product with B-type dye changed little. The fiber had a strong binding effect with the K-type dye, which changed the structure of the fiber. The main reason was the influence of high temperature. In Fig. 9d,e, compared with the infrared spectra of the unfixed samples, the two dye fixed samples showed that there was no significant change in the two maximum absorption peaks, indicating that the action of the fixing agent and the dye was moderate and did not affect the structure of the fibers.

## Conclusions

The optimum dyeing processes of soybean protein/flax blended yarns with the two types of reactive dyes were defined. For Reactive Yellow K-R, the optimum dye concentration was 2% owf., sodium chloride 40 g/L, sodium carbonate 10 g/L, fixation temperature 85 °C and time 30 min; For Reactive Yellow B-4RFN, the optimum dye concentration was 2% owf., sodium chloride 50 g/L, sodium carbonate 15 g/L, fixation temperature 70 °C for and time 50 min; in both the cases bath to material ratio was 20:1.

The application processes of formaldehyde-free color-fixing agent DM-2158 were also determined, namely, for blended yarns dyed with Reactive Yellow K-R: DM-2158 concentration 2% owf., bath to material ratio 10: 1, impregnated at impregnation temperature 45 °C for 20 min, cured at 115 °C for 1.5 min; for blended yarns dyed with Reactive Yellow B-4RFN: DM-2158 concentration 1.5% owf., bath to material ratio 10: 1, impregnation temperature 45 °C for 20 min and cured at 115 °C for 1 min.

The reproducibility of dyeing was good, and the colorfastness of the dyed samples could meet the general requirements, and B-type reactive dyes containing bifunctional groups showed a higher fixation level than that of K-type reactive dyes containing monofunctional group. The application of formaldehyde-free fixing agent to dyed products helped to improve colorfastness to washing and rubbing, especially for blended yarns dyed with K-type reactive dyes.
